# Food environment intervention improves food knowledge, wellbeing and dietary habits in primary school children: Project Daire, a randomised-controlled, factorial design cluster trial

**DOI:** 10.1186/s12966-021-01086-y

**Published:** 2021-02-04

**Authors:** Sarah F. Brennan, Fiona Lavelle, Sarah E. Moore, Moira Dean, Michelle C. McKinley, Patrick McCole, Ruth F. Hunter, Laura Dunne, Niamh E. O’Connell, Chris R. Cardwell, Chris T. Elliott, Danielle McCarthy, Jayne V. Woodside

**Affiliations:** 1 Institute for Global Food Security, Queen’s University Belfast, Belfast, BT9 5AG UK; 2 Centre for Public Health, Queen’s University Belfast, Belfast, BT12 6BA UK; 3 Centre for Evidence and Social Innovation, Queen’s University Belfast, Belfast, UK; 4 Queen’s Management School, Queen’s University Belfast, Belfast, BT9 5EE UK

**Keywords:** School, Children, Diet, Food, Education, Childhood wellbeing, Child behaviour, Food environment, Whole-school approach

## Abstract

**Background:**

Evidence suggests that dietary intake of UK children is suboptimal. As schools provide an ideal natural environment for public health interventions, effective and sustainable methods of improving food knowledge and dietary habits in this population must be identified. Project Daire aimed to improve children’s health-related quality of life, wellbeing, food knowledge and dietary habits via two multi-component interventions.

**Methods:**

Daire was a randomised-controlled, factorial design trial evaluating two interventions across four arms. Primary schools in Northern Ireland were randomised to one of four 6-month intervention arms: i) ‘Nourish’, ii) ‘Engage’, iii) ‘Nourish’ and ‘Engage’ and iv) Control (Delayed). ‘Nourish’ was an intervention aiming to alter the whole-school food environment, provide food-related experiences and exposure to locally produced foods. ‘Engage’ was an age-appropriate, cross-curricular educational intervention on food, agriculture, nutrition science and related careers. Primary outcomes were emotional and behavioural wellbeing and health-related quality of life. A number of secondary outcomes, including dietary intake, cooking competence and food-related knowledge, were also measured.

**Results:**

Fifteen schools from areas of varying socio-economic status participated in the randomised trial. A total of 903 (*n* = 445 aged 6–7 years and *n* = 458 aged 10–11 years) primary school pupils took part. Total Difficulties Score improved in all pupils (6–7 and 10–11 year old pupils) who received the ‘Nourish’ intervention compared with those that did not (adjusted difference in mean = − 0.82; 95% CI -1.46, − 0.17; *P* < 0.02). No statistically significant difference in Health-Related Quality of Life was observed. The ‘Nourish’ intervention also produced some changes in school-based dietary behaviour, which were most apparent in the 10–11 year old pupils. The ‘Nourish’ intervention also produced improvements in understanding of food labels (adjusted difference in mean = 0.15; 95% CI 0.05, 0.25; *P* < 0.01) and knowledge of vegetables in season (adjusted difference in mean = 0.29; 95% CI 0.01,0.56; *P* = 0.04) whilst an increased willingness to try new foods and improved perceived cooking competence was also observed.

**Conclusions:**

Improvements in childhood emotional and behavioural wellbeing, dietary intake, knowledge about food, cooking skills and willingness to try new foods were associated with the ‘Nourish’ whole-school food environment intervention. Exploration of the sustainability and long-term effectiveness of such whole-school food interventions should be conducted.

**Trial registration:**

National Institute of Health (NIH) U.S. National Library of Medicine Clinical Trials.gov (ID: NCT04277312).

**Supplementary Information:**

The online version contains supplementary material available at 10.1186/s12966-021-01086-y.

## Background

Poor diet quality in childhood has the potential to not only increase the risk of obesity and poor mental health in the short term [1, 2] but can also track into adulthood, which may increase risk of non-communicable disease in later life [1, 2]. Data published from the most recent UK National Diet and Nutrition Survey highlighted that dietary habits of UK school-aged children continue to be suboptimal [3, 4]. The data also suggest that dietary quality in childhood varies by household socio-economic status, highlighting the need to address health inequalities in early life [3].

Schools are ideal natural environments for public health interventions [5, 6]. As such, focus has shifted to the development of school food standards, such as those implemented across the UK in recent years, to try to improve the nutritional quality of school food [7]. In addition to this, the need for a ‘whole-school’ food approach, incorporating food-related education of children in addition to school food environment changes, has been highlighted [7]. The Health Promoting Schools (HPS) framework is a ‘whole-school’ approach advocated internationally to change lifestyle and health related behaviours in schools [6, 8]. However, lack of awareness and promotion of the HPS framework and poor understanding of the complexity of school systems have been identified as barriers to its implementation and evaluation [6].

Evidence from several systematic reviews of school-based nutrition education interventions has supported the effectiveness of a ‘whole-school’ approach, especially with regards to multi-component interventions delivered and supported by school staff and parents [9, 10]. However, more work needs to be done in terms of the development, implementation and evaluation of these interventions in varying contexts and regions, especially in the UK primary school setting where evidence is currently lacking. Project Daire undertook a multi-stakeholder approach to improve primary school children’s knowledge of, and interest in, food to improve health-related quality of life and wellbeing via two 6-month multi-component interventions. The interventions incorporated both whole-school food environment changes and food-related education and covered all food groups. Schools in areas of socio-economic disadvantage were targeted to address potential health inequalities. This paper reports on the primary outcomes of the trial and a number of secondary outcomes.

## Methods

### Study design

Daire was a randomised-controlled, factorial design four-arm trial evaluating two interventions in the primary school setting. Schools were randomised to one of four 6-month intervention arms: i) ‘Nourish’, ii) ‘Engage’, iii) ‘Nourish’ and ‘Engage’ and iv) Control (Delayed) and data were collected both pre and post-intervention. Project Daire worked in partnership with primary schools and a range of stakeholders, including teachers, principals, school caterers and local food producers, to develop interventions for pupils in year groups aged 6–7 and 10–11 years to improve knowledge of, and interest in, food. Primary schools were recruited from the North West of Northern Ireland to target a region of socio-economic disadvantage [11]. A range of schools from areas of varying socioeconomic status were recruited from both urban and rural locations. The study followed the Consolidated Standards of Reporting Trials (CONSORT) Statement [12] and is registered with National Institute of Health (NIH) U.S. National Library of Medicine Clinical Trials.gov (ID: NCT04277312). The CONSORT checklist for cluster trials is available as Additional File 1. Ethical approval was obtained from The School of Social Sciences, Education and Social Work Ethics Committee, Queen’s University Belfast (Reference number 038_1819).

### Recruitment

Mainstream (non-special) schools were eligible to participate if they met the following eligibility criteria: schools willing to be randomly assigned to an intervention, schools willing to engage with the intervention and implement it with their pupils, schools willing to facilitate data collection in their setting and schools located within the North West region of Northern Ireland. A list of all mainstream primary schools in the region was obtained from the local council and schools were contacted and informed about the study (*n* = 146). Thirty schools expressed interest in taking part and the first eligible schools to respond were sent a Memorandum of Understanding (MoU) which provided further details on the research. After being given time to consider the MoU, school principals or other nominated school contacts were asked to sign and return the MoU to the research team if they were willing to participate. Recruitment of schools took place between September 2018 and December 2018.

Pupils from participating schools were eligible to participate if they were in the class groups aged 6–7 years or 10–11 years during the academic year September 2018–June 2019. A system of opt-out parental consent was implemented. Participating schools were asked to distribute an information sheet to all parents of the relevant pupils which provided details on the purpose and aims of the study, the randomisation procedure, the interventions and the data collection process. Parents were given at least 48 h to consider the information and if parents did not want their child to participate in the data collection for the study, they were asked to complete and return the opt-out consent form. Consent was therefore assumed for all other pupils whose parents did not return completed opt-out forms.

### Interventions

Two interventions, ‘Nourish’ and ‘Engage’, were developed for both the 6–7 and 10–11 year old age groups. Intervention components are further detailed within the TIDieR Checklist, which is available within Additional File 2. The logic model which informed our intervention development is available within Additional File 7.

The ‘Nourish’ intervention was a whole-school food intervention aiming to alter the current school food environment of participating schools, promote a varied diet based on the four major food groups, provide food-related experiences and increase exposure to local Northern Ireland-sourced food. The ‘Nourish’ intervention aimed to influence childhood awareness of food across food groups, encourage tasting and identification of new foods, improve dietary intake and awareness of food preparation and cooking techniques. ‘Nourish’ was informed by pre-intervention observations conducted at schools (*n* = 11) in the region which captured information on current practices with the school food environment such as canteen protocols and systems, proportion of children who consumed school dinners versus packed lunches, school food provision and equipment and food-related events. These observations were conducted with input from school senior management and catering personnel. Specifically, the ‘Nourish’ intervention included provision of: healthy snacks e.g. fruit, a rotation of breads (including wheaten bread and high fibre bread with accompanying butter for spreading) and milk which were supplied by food industry partners during the school day; resources to improve school food presentation, cookery equipment and recipes which included all food groups; sensory education material; catering for school events; and attendance at Tasting Days at local Higher Education colleges to encourage tasting of locally produced foods. The ‘Nourish’ intervention also involved holding discussions with relevant school staff to help support the implementation of school food policies. Schools received a document highlighting relevant Public Health Agency Northern Ireland guidance [13] that would support them to implement their school food policies.

The ‘Engage’ intervention was an age-appropriate, cross-curricular educational intervention on food, agriculture, food and nutrition-related science and related careers. ‘Engage’ included topics such as the food chain, product development, growing food, animal welfare, sustainability, food labels, portion size and diet and health. The ‘Engage’ intervention incorporated aspects of the current Northern Ireland Curriculum, including literacy, mathematics and physical activity, and was designed to be flexible to enable easy implementation across schools [14]. Specifically, the ‘Engage’ intervention aimed to improve childhood knowledge about food across all food groups, awareness of preparation and cooking of food, the importance of a healthy diet and dietary intake. The intervention was developed by the research team in conjunction with stakeholders, including primary school teachers and local food producers who helped advise on type of content, integration of content within the current curriculum and tailoring of content to ensure age-appropriate The activities developed to support the lesson plans included videos, books, worksheets, games, talks/visits from visiting experts, visits to food producers, farm visit to school grounds, and practical experiments. The lesson plans and resources were supplied electronically and in hard copy and the intervention was largely delivered by teachers.

A listing of all available components for both interventions is available within Additional File 3. Intervention resources are available and can be requested by contacting study authors.

Schools allocated to ‘Nourish and Engage’ received both the ‘Nourish’ and ‘Engage’ interventions combined. Schools allocated to the control arm of study were offered the delayed ‘Engage’ intervention resources at the end of endpoint data collection for use during the following academic year and £500 for school funds.

### Randomisation and allocation

Schools were randomised to ‘Nourish’, ‘Engage’, ‘Nourish and Engage’ and Control (Delayed) arms following recruitment and baseline data collection. The allocation sequence was produced in STATA using block sizes of *n* = 4. Schools were stratified by religious affiliation, as this is how schools are typically organised with the Northern Ireland school system, to ensure a balanced approach. Three Irish language schools expressed interest in taking part but, due to study timeframes, it was not possible to translate all ‘Engage’ intervention resources in the event that these schools were randomised to an ‘Engage’ intervention arm. As such, the Irish language schools were not randomised and received the ‘Nourish’ intervention and data were collected (using translated outcome data collection questionnaires) and presented separately (Additional File 4). Therefore, Irish language schools are not presented in the main analyses.

### Data collection

Data were collected at baseline (February–March 2019) and endpoint (May–June 2019) by a team of researchers. The research team made arrangements with each school to attend for data collection during one school day at each time point. Teachers and research staff were present at all times during data collection to assist pupils with completion of the questionnaires. Data were collected via online survey and hard copy questionnaires. It was not possible to blind researchers to the intervention due to the nature of the data collection and intervention delivery process. However, the statistician who conducted data analyses was blinded to intervention allocation.

### Outcome measures

Outcomes measures were selected to address the related domains of childhood health, education and wellbeing which the interventions targeted [6]. This paper reports on primary outcomes and a number of secondary outcomes which were most directly related to the main aims of the interventions. Primary outcomes were emotional and behavioural wellbeing measured using the Strengths and Difficulties Questionnaire (SDQ) [15] and childhood health-related quality of life measured using the KIDSCREEN-10 questionnaire [16]. The primary outcome measures selected are designed for use by all primary school age children so were suitable for both the 6–7 and 10–11 year old age groups in the current study. As such, primary outcomes results were presented for all pupils together.

A number of secondary outcomes, which were most directly related to the mains aims of both interventions are also presented, including dietary intake measured via Food Frequency Questionnaire (FFQ), Agri-Food Knowledge score, Food Identification and Neophilia and Perceived Cooking Competence. Information on all outcomes reported in the present paper, including their development, administration and scoring, are detailed in Table 1. As detailed in Table 1, secondary outcomes were amended or developed specifically for the two different age groups under the guidance of primary school teachers. As such, questions were presented differently for both the 6–7 and 10–11 year old age groups and results presented separately. An outline of the other secondary outcomes not included in the current paper are presented within Additional File 5. These outcomes will be published separately.

**Table 1:** Study Outcomes Presented in the Current Paper

Study Outcomes	Information and Development	Administration	Scoring
KIDSCREEN-10	Widely used Health-Related Quality of Life measure validated in primary school age group [17]. KIDSCREEN-10 is a 10-item questionnaire with each item answered on a 5-point response scale. Items explore childhood physical activity and energy levels, emotions, depressive moods, stress, ability to enjoy recreational activities, socialising, relationships with parents/carers and other children and perception of cognitive capacity and school performance.	Both the 6–7 and 10–11 year-old pupil age groups were administered the same questionnaire at baseline and endpoint.	Raw scores were recoded so that higher values represented better health-related quality of life as per the KIDCREEN-10 manual [17] and summed. Syntax available from the questionnaire development team transformed scores to Rasch person parameters and T-values with means of 50 and a standard deviation of approximately 10.
Strengths and Difficulties (SDQ) Questionnaire	Validated childhood behaviour measure [15]. The version of the SDQ used in the present study is designed to be completed by parents or teachers of 4–16 year-old children. The SDQ consists of 5 scales (Emotional Problems Scale, Conduct Problems Scale, Hyperactivity Scale, Peer Problems Scale and ProSocial Scale) with each scale consisting of 5 items or questions. All scales represent a negative trait, with the exception of the ProSocial Scale which indicates a positive trait.	Teachers of the participating 6–7 and 10–11 year old pupils completed the SDQ for each child in their class at baseline and endpoint. The same questionnaire was completed by teachers of both the 6–7 and 10–11 year old age groups.	A scoring system is available for each individual scale via the questionnaire website (www.sdqinfo.com). The Total Difficulties Scale was calculated in addition to the component scores. Higher scores indicated higher levels of difficulty with the exception of the Pro Social Scale which is a positive trait. The Total Difficulties Scale is the sum of all component scores except the ProSocial Scale. The resultant score ranges from 0 to 40 and is counted as missing if one of the 4 component scores is missing.
Food Frequency Questionnaire (FFQ)	FFQ was developed by the study team and is based on another similar age-appropriate FFQ [18] which was validated in UK primary school children aged 3–7 years. The original FFQ was designed to be administered and used prospectively as a tick list record for all foods consumed over one 24-h period, with assistance from canteen staff/teachers and parents. For the purposes of the Daire project, the FFQ reference period was amended to ‘ever eaten’ any of the foods from the food list with optional responses of ‘yes’, ‘no’ and ‘not sure’. Researchers were present and able to assist children with completion of the FFQ during the data collection days.	10–11 year old pupils were administered two 52-item food lists, one referring to dietary intake at home, the other referring to dietary intake at school. A condensed 29-item food list was developed for the 6–7 year old pupils referring to ‘any/ever consumption’. FFQ was administered at baseline and endpoint.	For the purposes of analyses, the FFQ responses ‘no’ and ‘not sure’ were combined and compared with ‘yes’ responses. A selection of 11 foods from the 6–7 year old 30-item food list and 18 foods from the 105-item 10–11 year old food list were included in analyses to represent foods that the children were exposed to via the Nourish or Engage interventions
Agri-Food Knowledge	An Agri-Food Knowledge measure was developed based on input from industry partners, the British Nutrition Foundation-Food a Fact of Life resource [19] and adapted from previously published measures [20, 21].	6–7 and 10–11 year old pupils at baseline and endpoint. A shorter measure was administered to 6–7 year old pupils	The measure consisted of a series of component scores including a farm knowledge score, food chain knowledge score, science relating to food knowledge score, local versus imported knowledge score, knowledge of vegetables in season, product to source knowledge score, food label knowledge score. A total Agri-Food score was calculated. Higher score indicated better agri-food related knowledge.
Food Identification and Food Neophilia	A measure assessing ability to identify a range of foods including vegetables, salmon, bread and willingness to try these foods was adapted from previously published measures [22, 23].	6–7 and 10–11 year old pupils at baseline and endpoint. A shorter measure was administered to 6–7 year old children.	Measure was scored as one point for a correct response and willingness to taste these foods.
Perceived Cooking Competence	Measure based on other similar measures for perceived movement competence [24, 25] and an adult cooking confidence measure previously developed by the study team [26].	10–11 year old pupils at baseline and endpoint. This measure was intended for both age groups, with a reduced number of items for the 6–7 year olds. As internal consistency was not adequate for the younger group with reduced items, results are presented for 10–11 year old group only.	Children rated their performance of a range of cooking skills from 1 to 5 using child-friendly options. Higher score indicated better perceived cooking competence.

### Sample size

A sample size calculation was conducted to determine the differences the study would be able to detect as statistically significant. The power calculations in this factorial design intervention compared ‘Nourish’ versus not Nourish and ‘Engage’ versus not Engage. The study was not powered to test for an interaction between ‘Nourish’ and ‘Engage’. Assuming 12 schools completed the intervention (6 in the intervention and 6 in the control group), and with a maximum of 40 pupils per class (Primary 3 or Primary 7) in each school, based upon a standard deviation of the primary outcome measure (Strengths and Difficulties Questionnaire-SDQ) of 4.5 from [27] and using ICC within schools of 0.1 [28, 29] based upon the ICCs used in a previous calculation or ICCs for similar outcomes, the study would have had over 80% power to detect as statistically significant at the 5% level a difference in SDQ of 2.8 units. For the other primary outcome measure, the KIDSCREEN-10, the detectable difference calculated using a similar calculation was 5.6, based upon a standard deviation of the KIDSCREEN-10 of 9.0. Therefore, the study required a total of 12 schools with an average of 40 children aged 6–7 years and 40 aged 10–11 years, i.e. 80 × 12 = 960 pupils in total. In order to account for potential drop-out, or the possibility that some school would have smaller year groups, a generous 20% drop-out rate was applied. As such, the aim was to recruit a total of 1152 pupils.

### Compliance and retention

School recruitment and retention was monitored. To maximise completion of data collection at each study time point, pupils received a token of appreciation at the end of data collection and teachers received a small gift voucher worth £10. Intervention fidelity will be examined as part of process evaluation and will be presented separately.

### Statistical analyses

The primary analysis of continuous variables (such as the primary outcomes: Strengths and Difficulties Questionnaire and KIDSCREEN-10) was conducted using linear regression with the endpoint value as the outcome and both group variables (‘Nourish’ and ‘Engage’) and baseline value included in the model. Furthermore, robust standard errors [30] were used to account for lack of independence of children within a school (implemented using the cluster command in STATA). Consequently, the difference in mean of the outcome, with 95% confidence intervals (95% CIs) was calculated, in the Nourish/Engage versus the non-Nourish/Engage group adjusting for baseline and accounting for clustering. Primary outcomes were examined by gender. A similar analysis was conducted for binary outcomes at the endpoint using logistic regression to compare the intervention groups adjusting for clustering, though not adjusting for outcomes at baseline. In this analysis odds ratios (ORs) and 95% CIs were calculated to compare Nourish/Engage versus the non-Nourish/Engage group [31]. Analyses were conducted using STATA (Version 15, StataCorp LCC, College Station, TX) and SPSS for Windows Version 26 (IBM Corp., Armonk, N.Y., USA).

## Results

**Fig. 1:**
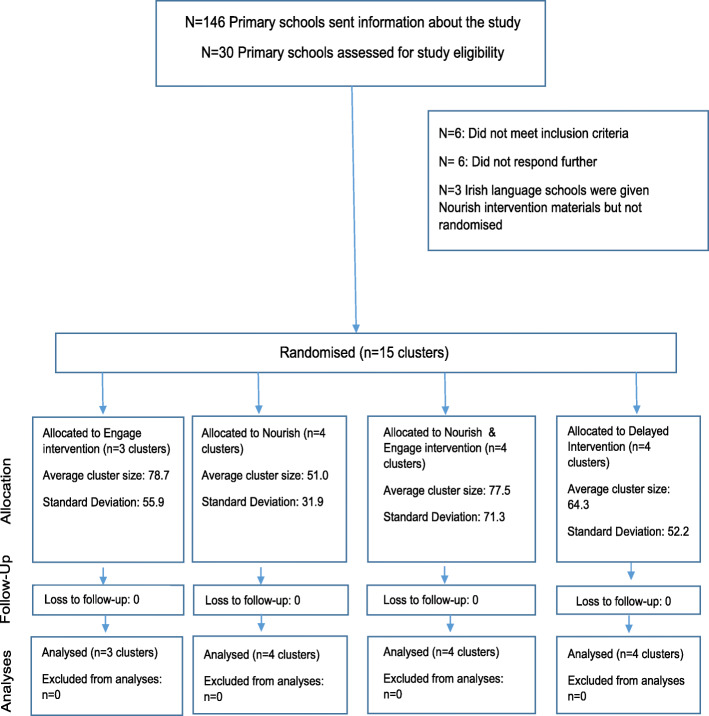
CONSORT Cluster Trials Flow Diagram showing Progression of Schools through the Study

In total, 30 schools were assessed for study eligibility. Six schools did not meet inclusion criteria, six schools did not respond further and three Irish language schools were not randomised but were directly allocated to the ‘Nourish’ intervention, as previously described. In total, 15 primary schools were randomised in the present study. From these schools, 25 classes of 6–7 year-old pupils and 22 classes of 10–11 year old pupils participated in the study. All 15 schools completed the study. Parental consent was obtained for 903 pupils. A CONSORT flow diagram presenting flow of clusters through the study is shown in Fig. 1. School characteristics are presented in Table 2.

**Table 2:** School and Pupil Characteristics by Project Daire Study Intervention Arm

	Nourish	No Nourish	Engage	No Engage
**Number of Schools**	8	7	7	8
**Rural**	4	1	1	4
**Urban**	4	6	6	4
**6–7 year old pupils**	320 (54.1)	230 (49.3)	277 (52.2)	273 (51.7)
**10–11 year old pupils**	272 (45.9)	237 (50.7)	254 (47.8)	255 (48.3)
**Male**	297 (50.2)	236 (50.5)	253 (47.6)	280 (53.0)
**Female**	295 (49.8)	231 (49.5)	278 (52.4)	248 (47.0)

All schools randomised to Nourish (100%) received recipe books to take home, equipment to run cookery activities, a flavour school sensory resource, healthy eating policy recommendations and equipment to enhance school food presentation in canteens (posters, tablecloths and bunting). All schools (100%) randomised to Nourish also received weekly snacks for the pupils (fruit, milk or bread) provided by industry partners. All but one (88.8%) of the schools randomised to Nourish attended a Tasting Day as part of the intervention which was held at a local Higher Education College. Two of the schools (22.2%) randomised to Nourish organised an optional catered event, with food provided by industry partners.

All schools randomised to Engage (100%) received educational activities and resources for sixteen lessons over three topic areas specific to both participating year groups. This included pre-defined learning intentions, lesson plans and resources to support lesson delivery such as worksheets, videos, games, and practical activities. Five lessons (with associated activities, such as storybooks for the pupils, seeds for growing and one talk from an in-person class visitor) were suggested as core content, although teachers were encouraged to deliver as much of the content as possible. All schools (100%) randomised to the Engage intervention availed of the optional visiting speakers and a visiting farm to support the lessons. Schools randomised to Engage also had the opportunity to visit food production facilities which two (28.5%) of the schools availed of.

Qualitative feedback received from teachers indicated that pupils especially enjoyed the snack provision and Tasting Days as part of the Nourish intervention and multiple teachers reported pupils were more open to trying new foods after these activities. Feedback received from teachers also indicated that they found the lesson resources provided as part of the Engage intervention very useful and that they especially liked the externally delivered visiting speakers (including the food based physical education class). Teachers did report, whether on Nourish or Engage, that they would prefer to utilise the resources provided over two terms rather than one term due to time pressures and timetable planning.

 Results from both of the primary outcomes, the Strengths and Difficulties Questionnaire and the KIDSCREEN-10, are presented in Table 3 for all randomised pupils. In the SDQ, the mean Total Difficulties Score significantly reduced in the ‘Nourish’ group from 7.82 at baseline to 6.86 at endpoint but did not change significantly in those who did not receive the ‘Nourish’ intervention (baseline 7.65 to endpoint 7.54). This corresponded to an overall reduction in Total Difficulties Score at endpoint of − 0.82 in the ‘Nourish’ group versus those that did not receive the ‘Nourish’ intervention (adjusted difference in mean = − 0.82 95% CI-1.46,-0.17; *P* < 0.02). Similarly, the component Conduct Problem Score (adjusted difference in mean = − 0.19 95% CI -0.37, − 0.01; *P* = 0.04) also reduced significantly in those that received the ‘Nourish’ intervention versus those that did not receive the ‘Nourish’ intervention. No significant differences in SDQ scores were observed between those who received the ‘Engage’ intervention and those that did not receive the ‘Engage’ intervention. No significant differences in the Health-Related Quality of Life scores from the KIDSCREEN-10 questionnaire were observed in those that received either the ‘Nourish’ or ‘Engage’ interventions compared with those that did not receive either intervention.

**Table 3:** Impact of the Nourish and Engage Interventions on Strengths and Difficulties (SDQ) and KIDSCREEN-10 Rasch Parameter Estimates and International T-Values (All age groups)

	Nourish	No Nourish	Engage	No Engage
**Emotional Problems (SDQ)**				
**N (baseline and follow-up responses)**	279	254	322	211
**Baseline Mean (SD)**	2.05 (2.54)	2.08 (2.58)	2.18 (2.67)	1.90 (2.36)
**Follow-up Mean (SD)**	1.67 (2.27)	1.97 (2.50)	2.01 (2.52)	1.51 (2.12)
**Adjusted diff. in mean (95% CI)**	−0.29 (− 0.65–0.07)	Reference	0.33 (− 0.00–0.65)	Reference
***P*-value**	0.10		0.05	
**Conduct Problems Scale (SDQ)**				
**N (baseline and follow-up responses)**	278	255	319	214
**Baseline Mean (SD)**	1.15 (1.96)	1.19 (1.99)	1.17 (1.99)	1.16 (1.96)
**Follow-up Mean (SD)**	1.02 (1.77)	1.23 (1.95)	1.06 (1.82)	1.22 (1.91)
**Adjusted diff. in mean (95% CI)**	−0.19 (−0.37- -0.01)	Reference	−0.15 (− 0.32–0.02)	Reference
**P-value**	0.04		0.08	
**Hyperactivity Scale (SDQ)**				
**N (baseline and follow-up responses)**	281	260	325	216
**Baseline Mean (SD)**	3.32 (1.96)	3.09 (3.14)	3.14 (1.98)	3.31 (3.26)
**Follow-up Mean (SD)**	3.11 (3.12)	3.02 (3.12)	2.92 (3.09)	3.29 (3.15)
**Adjusted diff. in mean (95% CI)**	−0.08 (−0.33–0.17)	Reference	−0.23 (− 0.48–0.02)	Reference
***P*-value**	0.48		0.07	
**Peer Problems Scale (SDQ)**				
**N (baseline and follow-up responses)**	279	256	325	210
**Baseline Mean (SD)**	1.29 (1.75)	1.29 (1.64)	1.32 (1.63)	1.23 (1.79)
**Follow-up Mean (SD)**	1.03 (1.59)	1.25 (1.77)	1.12 (1.72)	1.16 (1.62)
**Adjusted diff. in mean (95% CI)**	−0.21 (−0.46–0.04)	Reference	−0.11 (− 0.34–0.12)	Reference
***P*-value**	0.09		0.33	
**ProSocial Scale (SDQ)**				
**N (baseline and follow-up responses)**	278	255	320	213
**Baseline Mean (SD)**	7.46 (2.65)	7.74 (2.50)	7.71 (2.68)	7.41 (2.41)
**Follow-up Mean (SD)**	7.59 (2.64)	7.88 (2.41)	7.82 (2.58)	7.59 (2.47)
**Adjusted diff. in mean 95% CI)**	−0.09 (−0.81–0.62)	Reference	0.03 (−0.65–0.70)	Reference
***P*-value**	0.77		0.93	
**Total Difficulties Score (SDQ)**				
**N (baseline and follow-up responses)**	274	246	316	204
**Baseline Mean (SD)**	7.82 (7.38)	7.65 (7.01)	7.81 (7.36)	7.64 (6.96)
**Follow-up Mean (SD)**	6.86 (6.65)	7.54 (6.94)	7.16 (6.96)	7.21 (6.55)
**Adjusted diff. in mean 95% CI)**	−0.82 (−1.46- -0.17)	Reference	−0.16 (− 0.76–0.44)	Reference
***P*-value**	0.02		0.58	
**General Health Related Quality of Life Index Rasch Parameter Estimates (KIDSCREEN-10)**				
**N (baseline and follow-up responses)**	402	381	222	172
**Baseline Mean (SD)**	1.08 (1.07)	1.07 (1.09)	1.11 (1.11)	1.09 (1.07)
**Follow-up Mean (SD)**	1.36 (1.16)	1.25 (1.16)	1.37 (1.18)	1.42 (1.10)
**Adjusted diff. in mean 95% CI)**	0.11 (−0.06–0.30)	Reference	−0.04 (− 0.21–0.14)	Reference
***P*-value**	0.18		0.64	
**General Health Related Quality of Life Index International T Values (KIDSCREEN-10)**				
**N (baseline and follow-up responses)**	402	381	222	172
**Baseline Mean (SD)**	51.51 (11.25)	48.68 (10.52)	51.61 (11.48)	52.09 (10.69)
**Follow-up Mean (SD)**	48.77 (10.37)	50.40 (11.26)	49.10 (10.76)	48.87 (10.37)
**Adjusted diff. in mean 95% CI)**	1.12 (−0.61–2.85)	Reference	−0.37 (−2.06–1.32)	Reference
***P*-value**	0.18		0.65	

*P* value < 0.05 indicative of significance; *N* Number; *SD* Standard Deviation. In factorial analysis, the 2 main effects (Nourish compared with no nourish, and Engage compared with no engage) are investigated

Gender analyses were conducted and results suggest that the improvement in Total Difficulties in those randomised to the Nourish intervention was driven by male pupils, as Total Difficulties score improved in males who received Nourish compared with those who did not (− 0.75; 95% CI: − 1.36, -0.12; *P* = 0.02). Furthermore, significant improvements in health-related quality of life were observed in males randomised to the Nourish intervention were observed compared with male pupils who did not receive Nourish (KIDSCREEN-10 General Health Related Quality of Life Index Rasch Parameter Estimate adjusted difference in mean = 0.23; 95% CI 0.13, 0.45; *P* = 0.03 and KIDSCREEN-10 General Health Related Quality of Life Index International T Values adjusted difference in mean = 2.31; 95% CI: 0.13, 4.47; *P* = 0.03) (Additional File 6).

Please note when the Irish language schools were included in additional analyses, the Conduct Problem score (adjusted difference in mean = − 0.27 95% CI -0.54, − 0.00; *P* = 0.04) also significantly reduced in those that received the ‘Nourish’ intervention versus those that did not receive the ‘Nourish’ intervention. A reduction in Total Difficulties Score was observed which approached significance (*p* = 0.05). For more detailed results, see Additional File 4.

Age-specific results from the FFQ analyses are presented in Table 4. Pupils aged 6–7 years were asked about ‘ever’ or ‘never’ consumption of the FFQ food items. In the 6–7 year old group, 86.0% of pupils who received the ‘Nourish’ intervention at endpoint ever consumed vegetables compared with 71.4% in those who did not receive ‘Nourish’, corresponding to an Odds Ratio of 2.42 (95% CI 1.63, 3.59; *P* < 0.01). Pupils aged 6–7 years old who received ‘Nourish’ were less likely to ever consume beef compared with those that did not (*p* < 0.01). Indications of dietary change were, however, most apparent in 10–11 year old pupils with regards to school dietary intake in those that received the ‘Nourish’ intervention. Pupils aged 10–11 years were asked about their consumption of the FFQ food items at school and at home separately. Pupils who received the Nourish intervention were more likely to consume apples (*p* = 0.008), mushrooms (*p* = 0.002), white (*p* = 0.01) and brown/wholemeal bread (*p* < 0.001), milk to drink (*p* = 0.004), chicken (*p* < 0.001) and bacon/ham (*p* < 0.001) post-intervention compared with 10–11 year old pupils that did not receive this intervention. These pupils were also, however, more likely to consume chocolate (*p* < 0.001) and fizzy drinks (*p* = 0.001).

**Table 4:** Impact of the Nourish and Engage Interventions on any food intake of 6–7 year old pupils and school food intake in 10–11 year old pupils at Endpoint measured using FFQ

All Food Intake in 6–7 year old pupils	Nourish	No Nourish	Engage	No Engage	School Food Intake in 10–11 year old pupils	Nourish	No Nourish	Engage	No Engage
**Fruit n** Pupils eating food Endpoint n(%) Treatment effect (OR 95% CI) *P*-value	231 225 (97.4) 2.30 (0.92–5.74) 0.08	205 193 (94.2) Reference-	242 233 (96.3) 1.10 (0.45–2.69) 0.84	194 185 (95.4) Reference -	**Apples n** Pupils eating food Endpoint n(%) Treatment effect (OR 95% CI) *P*-value	196 171 (87.2) 1.27 (1.06–1.53) 0.008	195 166 (85.1) Reference -	212 177 (83.5) 0.58 (0.47–0.72) 0.000	179 160 (89.4) Reference -
					**Grapes n** Pupils eating food Endpoint n(%) Treatment effect (OR 95% CI) *P*-value	194 146 (75.3) 1.55 (0.80–3.02) 0.19	193 127 (65.8) Reference -	210 152 (72.4) 1.15 (0.56–2.38) 0.70	177 121 (68.4) Reference -
					**Bananas n** Pupils eating food Endpoint n(%) Treatment effect (OR 95% CI) *P*-value	190 128 (67.4) 1.15 (0.83–1.60) 0.40	194 124 (63.9) Reference -	210 140 (66.7) 1.09 (0.79–1.50) 0.61	174 112 (64.4) Reference -
					**Strawberries n** Pupils eating food Endpoint n(%) Treatment effect (OR 95% CI) *P*-value	194 134 (69.1) 1.64 (0.94–2.87) 0.08	192 108 (56.3) Reference -	210 145 (69.1) 1.73 (0.98–3.04) 0.06	176 97 (55.1) Reference -
**Vegetables n** Pupils eating food Endpoint n(%) Treatment effect (OR 95% CI) *P*-value	228 196 (86.0) 2.42 (1.63–3.59) 0.000	203 145 (71.4) Reference -	239 193 (80.8) 1.07 (0.72–1.58) 0.75	`192 148 (77.1) Reference -	**Vegetables n** Pupils eating food Endpoint n(%) Treatment effect (OR 95% CI) *P*-value	183 149 (81.4) 1.19 (0.69–2.04) 0.53	190 150 (79.0) Reference -	204 162 (79.4) 0.88 (0.50–1.56) 0.66	169 137 (81.1) Reference -
					**Mushrooms n** Pupils eating food Endpoint n(%) Treatment effect (OR 95% CI) *P*-value	192 24 (12.5) 2.29 (1.35–3.91) 0.002	194 11 (5.7) Reference -	209 22 (10.5) 1.35 (0.78–2.33) 0.29	177 13 (7.3) Reference -
**White or wholemeal bread** Pupils eating food Endpoint n(%) Treatment effect (OR 95% CI) *P*-value	232 215 (92.7) 0.65 (0.28–1.48) 0.30	203 193 (95.1) Reference -	243 228 (93.8) 1.09 (0.45–2.67) 0.85	192 180 (93.8) Reference -	**White bread n** Pupils eating food Endpoint n(%) Treatment effect (OR 95% CI) *P*-value	194 164 (84.5) 1.83 (1.15–2.92) 0.01	200 149 (74.5) Reference -	213 173 (81.2) 1.18 (0.69–2.01) 0.55	181 140 (77.4) Reference -
					**Brown/wholemeal bread n** Pupils eating food Endpoint n(%) Treatment effect (OR 95% CI) *P*-value	194 128 (66.0) 2.22 (1.51–3.26) 0.000	198 90 (45.5) Reference -	212 131 (61.8) 1.60 (1.07–2.38) 0.02	180 87 (48.3) Reference -
**Pancakes/ scones/ fruit bread n** Pupils eating food Endpoint n(%) Treatment effect (OR 95% CI) *P*-value	228 210 (92.1) 1.56 (0.78–3.10) 0.21	205 181 (88.3) Reference -	240 217 (90.4) 0.96 (0.50–1.83) 0.90	193 174 (90.2) Reference -	**Pancakes/ scones/ fruit bread n** Pupils eating food Endpoint n(%) Treatment effect (OR 95% CI) *P*-value	189 113 (59.8) 0.92 (0.55–1.52) 0.74	196 121 (61.7) Reference -	205 125 (61.0) 1.03 (0.61–1.73) 0.91	180 109 (60.6) Reference -
**Milk to drink/ on cereal n** Pupils eating food Endpoint n(%) Treatment effect (OR 95% CI) *P*-value	227 208 (91.6) 1.02 (0.45–2.29) 0.97	204 187 (91.7) Reference -	239 218 (91.2) 0.88 (0.40–1.93) 0.75	192 177 (92.2) Reference -	**Milk to drink n** Pupils eating food Endpoint n(%) Treatment effect (OR 95% CI) *P*-value	190 130 (68.4) 1.66 (1.18–2.33) 0.004	196 111 (56.6) Reference -	206 130 (63.1) 1.00 (0.69–1.43) 0.98	180 111 (61.7) Reference -
					**Margarine/ Butter n** Pupils eating food Endpoint n(%) Treatment effect (OR 95% CI) *P*-value	192 128 (66.7) 1.45 (0.98–2.14) 0.07	199 113 (56.8) Reference -	211 141 (66.8) 1.54 (1.01–2.36) 0.045	180 100 (55.6) Reference -
**Chicken (sliced, no sauce) n** Pupils eating food Endpoint n(%) Treatment effect (OR 95% CI) *P*-value	221 138 (62.4) 0.71 (0.49–1.03) 0.07	206 145 (70.4) Reference -	236 153 (64.8) 0.91 (0.60–1.39) 0.67	191 130 (68.1) Reference -	**Chicken (sliced, no sauce) n** Pupils eating food Endpoint n(%) Treatment effect (OR 95% CI) *P*-value	192 120 (62.5) 2.45 (1.55–3.88) 0.000	197 82 (41.6) Reference -	210 104 (49.5) 0.71 (0.45–1.14) 0.15	179 98 (54.8) Reference -
**Beef n** Pupils eating food Endpoint n(%) Treatment effect (OR 95% CI) *P*-value	180 101 (56.1) 0.35 (0.28–0.43) 0.000	123 97 (78.9) Reference -	91 91 (61.9) 0.91 (0.74–1.11) 0.35	107 107 (68.6) Reference -	**Beef n** Pupils eating food Endpoint n(%) Treatment effect (OR 95% CI) *P*-value	224 32 (14.3) 1.27 (0.86–1.86) 0.22	235 36 (15.3) Reference -	251 40 (15.9) 1.15 (0.78–1.70) 0.47	208 28 (13.5) Reference -
**Bacon/ ham/ sausages n** Pupils eating food Endpoint n(%) Treatment effect (OR 95% CI) *P*-value	229 210 (91.7) 0.99 (0.52–1.91) 0.98	203 187 (92.1) Reference-	243 221 (91.0) 0.74 (0.34–1.62) 0.46	189 176 (93.1) Reference -	**Bacon/ ham n** Pupils eating food Endpoint n(%) Treatment effect (OR 95% CI) *P*-value	192 139 (72.4) 1.68 (1.29–2.18) 0.000	199 121 (60.8) Reference -	211 143 (67.8) 1.06 (0.83–1.37) 0.64	180 117 (65.0) Reference -
					**Sausages n** Pupils eating food Endpoint n(%) Treatment effect (OR 95% CI) *P*-value	194 147 (75.8) 1.11 (0.73–1.68) 0.64	199 147 (73.9) Reference -	212 159 (75.0) 1.01 (0.67–1.53) 0.97	181 135 (74.6) Reference -
**Fish fillet/ tuna n** Pupils eating food Endpoint n(%) Treatment effect (OR 95% CI) *P*-value	220 93 (42.3) 0.71 (0.44–1.15) 0.17	197 98 (49.8) Reference -	234 112 (47.9) 1.28 (0.77–2.12) 0.34	183 79 (43.2) Reference -	**Fish fillet n** Pupils eating food Endpoint n(%) Treatment effect (OR 95% CI) *P*-value	193 49 (25.4) 1.34 (0.84–2.16) 0.22	194 38 (19.6) Reference -	209 53 (25.4) 1.39 (0.85–2.27) 0.19	178 34 (19.1) Reference -
**Biscuits/ chocolate n** Pupils eating food Endpoint n(%) Treatment effect (OR 95% CI) *P*-value	229 216 (94.3) 1.79 (0.98–3.26) 0.06	204 186 (91.2) Reference -	243 222 (91.4) 0.53 (0.25–1.13) 0.10	190 180 (94.7) Reference -	**Chocolate n** Pupils eating food Endpoint n(%) Treatment effect (OR 95% CI) *P*-value	193 134 (69.4) 3.19 (1.69–6.03) 0.000	198 85 (42.9) Reference -	211 113 (53.6) 0.68 (0.37–1.26) 0.22	180 106 (58.9) Reference -
**Fizzy drink n** Pupils eating food Endpoint n(%) Treatment effect (OR 95% CI) *P*-value	232 190 (81.9) 0.88 (0.51–1.52) 0.65	204 171 (83.8) Reference -	244 201 (82.4) 0.96 (0.56–1.62) 0.87	192 160 (83.3) Reference -	**Fizzy drink n** Pupils eating food Endpoint n(%) Treatment effect (OR 95% CI) *P*-value	194 63 (32.5) 1.96 (1.32–2.89) 0.001	198 39 (19.7) Reference -	212 57 (26.9) 1.02 (0.66–1.56) 0.94	180 45 (25.0) Reference -

^a^n: Total number of pupils that answered questionnaire item at Endpoint data collection. In factorial analysis, the 2 main effects (Nourish compared with no nourish, and Engage compared with no engage) are investigated. *P* value < 0.05 indicative of significance,

Age-specific results from the Agri-Food and Component scores are presented in Table 5. Although there were no statistically significant effects on overall Agri-Food Knowledge Score, significant differences in some of its component scores were observed between those who received one of the interventions compared with those who did not. In 6–7 year old pupils who received the ‘Nourish’ intervention, understanding of food labels increased from 0.17 at baseline to 0.46 at endpoint but did not change significantly in the non-Nourish group from baseline (0.20) to endpoint (0.33). This corresponded to an improvement in understanding of food labels of 0.15 (adjusted difference in mean = 0.15; 95% CI 0.05,0.25; *P* < 0.01), whilst no significant effects associated with the ‘Engage’ intervention were observed. In 10–11 year-old pupils who received the ‘Nourish’ intervention compared with those who did not, their knowledge of vegetables in season significantly increased (adjusted difference in mean = 0.29; 95% CI 0.01,0.56; *P* = 0.04). In 10–11 year-old pupils who received the ‘Engage’ intervention compared with those who did not, their understanding of food labels significantly increased (adjusted difference in mean = 0.70 95% CI 0.30,1.10; *P* < 0.01).

**Table 5:** Impact of the Nourish and Engage Interventions on Agri-Food Score and Component Scores in both 6–7 and 10–11 year old Pupils

	6–7 year old pupils				10–11 year old pupils			
	Nourish	No Nourish	Engage	No Engage	Nourish	No Nourish	Engage	No Engage
**Farm Knowledge Score**								
**N (baseline and follow-up responses)**	217	198	225	190	188	183	195	176
**Baseline Mean (SD)**	2.19 (0.88)	2.13 (0.95)	2.12 (0.93)	2.22 (0.89)	6.15 (1.70)	5.81 (1.72)	5.95 (1.76)	6.03 (1.66)
**Follow-up Mean (SD)**	2.33 (0.82)	2.20 (0.93)	2.29 (0.86)	2.24 (0.90)	6.15 (1.66)	5.79 (1.89)	5.97 (1.70)	5.97 (1.88)
**Adjusted diff. in mean 95% CI)**	0.12 (−.02–0.26)	Reference	0.06 (−0.08–0.20)	Reference	0.24 (−.06–0.4)	Reference	− 0.00 (− 0.32–0.32)	Reference
***P*-value**	0.10		0.36		0.11		1.00	
**Food Chain Knowledge Score**								
**N (baseline and follow-up responses)**	218	198	225	191	238	237	259	216
**Baseline Mean (SD)**	0.90 (1.03)	0.54 (0.82)	0.76 (0.99)	0.70 (0.91)	2.05 (1.39)	1.75 (1.40)	1.82 (1.42)	2.00 (1.37)
**Follow-up Mean (SD)**	1.59 (1.20)	1.08 (1.16)	1.48 (1.23)	1.18 (1.16)	2.21 (1.49)	2.05 (1.39)	2.15 (1.45)	2.10 (1.45)
**Adjusted diff. in mean 95% CI)**	0.29 (−0.16–0.73)	Reference	0.23 (− 0.21–0.66)	Reference	0.03 (− 0.22–.28)	Reference	.13 (− 0.12–0.37)	Reference
***P*-value**	0.19		0.29		0.80		0.29	
**Science relating to Food Knowledge Score**								
**N (baseline and follow-up responses)**	217	198	225	190	188	183	195	176
**Baseline Mean (SD)**	1.52 (1.20)	1.46 (1.26)	1.56 (1.25)	1.42 (1.20)	3.11 (1.58)	3.00 (1.46)	3.12 (1.43)	2.98 (1.62)
**Follow-up Mean (SD)**	1.83 (1.24)	1.79 (1.19)	1.92 (1.18)	1.70 (1.24)	3.33 (1.63)	3.12 (1.67)	3.38 (1.53)	3.05 (1.78)
**Adjusted diff. in mean 95% CI)**	1.11 (−0.28–0.28)	Reference	0.21 (− 0.07–0.48)	Reference	0.13 (− 0.22–0.47)	Reference	.26 (− 0.07–0.59)	Reference
***P*-value**	1.00		0.13		0.44		0.12	
**Local V Imported Knowledge Score**								
**N (baseline and follow-up responses)**	217	198	225	190	188	183	195	176
**Baseline Mean (SD)**	5.62 (1.54)	5.03 (1.48)	5.17 (1.53)	5.53 (1.53)	7.67 (1.53)	7.17 (1.54)	7.31 (1.45)	7.55 (1.66)
**Follow-up Mean (SD)**	5.92 (1.63)	5.51 (1.46)	5.82 (1.56)	5.61 (1.57)	7.53 (1.62)	7.43 (1.56)	7.41 (1.56)	7.55 (1.63)
**Adjusted diff. in mean 95% CI)**	0.28 (− 0.23–0.80)	Reference	0.23 (− 0.26–0.72)	Reference	−0.11 (− 0.57–0.35)	Reference	−0.02 (− 0.47–0.43)	Reference
***P*-value**	0.26		0.33		0.61		0.93	
**Vegetables in Season Knowledge Score**								
**N (baseline and follow-up responses)**	218	198	225	191	188	183	195	176
**Baseline Mean (SD)**	0.91 (0.89)	0.89 (1.02)	0.90 (0.89)	0.91 (1.02)	1.08 (1.09)	1.22 (1.06)	1.03(1.03)	1.28 (1.11)
**Follow-up Mean (SD)**	0.96 (0.79)	0.94 (0.84)	0.94 (0.82)	0.96 (0.81)	1.38 (1.10)	1.13 (0.96)	1.22 (1.08)	1.30 (0.99)
**Adjusted diff. in mean 95% CI)**	0.02 (−0.18–0.22)	Reference	−0.01 (− 0.21–0.18)	Reference	0.29 (0.01–0.56)	Reference	−0.08 (− 0.36–0.19)	Reference
***P*-value**	0.84		0.89		0.04		0.54	
**Product To Source Knowledge Score**								
**N (baseline and follow-up responses)**	224	201	234	191	156	156	160	152
**Baseline Mean (SD)**	5.78 (1.79)	5.26 (1.91)	5.57 (1.84)	5.49 (1.90)	7.91 (1.30)	7.65 (1.68)	7.90 (1.43)	7.65 (1.57)
**Follow-up Mean (SD)**	5.73 (1.74)	5.68 (1.74)	5.69 (1.77)	5.72 (1.70)	7.82 (1.49)	7.81 (1.69)	7.78 (1.68)	7.85 (1.49)
**Adjusted diff. in mean 95% CI)**	−0.44 (− 0.38–0.29)	Reference	−0.04 (− 0.37–0.29)	Reference	−0.07 (− 0.38–0.23)	Reference	−0.16 (− 0.48–0.16)	Reference
***P*-value**	0.78		0.79		0.61		0.30	
**Food Label Knowledge Score**								
**N (baseline and follow-up responses)**	218	198	225	191	188	183	195	176
**Baseline Mean (SD)**	0.17 (0.42)	0.20 (0.42)	0.20 (0.44)	0.17 (0.39)	2.17 (1.23)	2.02 (1.26)	2.32 (1.23)	1.85 (1.22)
**Follow-up Mean (SD)**	0.46 (0.64)	0.33 (0.58)	0.40 (0.63)	0.40 (0.60)	2.86 (1.27)	2.54 (1.39)	3.14 (1.21)	2.22 (1.31)
**Adjusted diff. in mean 95% CI)**	0.15 (0.05–0.25)	Reference	−0.03 (−0.14–0.07)	Reference	0.16 (− 0.24–0.55)	Reference	0.70 (0.30–1.10)	Reference
***P*-value**	0.006		0.52		0.41		< 0.01	
**Total AgriFood Knowledge Score**								
**N (baseline and follow-up responses)**	254	224	269	209	188	183	195	176
**Baseline Mean (SD)**	16.09 (4.84)	14.38 (4.22)	15.07 (4.84)	15.57 (4.34)	29.80 (5.41)	28.25 (5.20)	29.02 (5.11)	29.05 (5.63)
**Follow-up Mean (SD)**	17.31 (5.25)	16.65 (4.04)	17.07 (5.10)	16.99 (4.22)	30.75 (5.95)	29.31 (6.19)	30.53 (5.95)	29.49 (6.24)
**Adjusted diff. in mean 95% CI)**	0.42 (−1.44–2.27)	Reference	.04 (−1.66–1.74)	Reference	0.39 (−1.46–2.24)	Reference	1.00 (−0.90–2.90)	Reference
***P*-value**	0.64		0.96		0.66		0.27	

*P* value < 0.05 indicative of significance; *N* Number; *SD* Standard Deviation. In factorial analysis, the 2 main effects (Nourish compared with no nourish, and Engage compared with no engage) are investigated

Age-specific results from the Food Identification, Food Neophilia and Perceived Cooking Competence Scores are presented in Table 6. Willingness to trying new foods significantly increased in 6–7 year old pupils who received the ‘Nourish’ intervention compared with those that did not (adjusted difference in mean = 0.27; 95% CI 0.03,0.51; *P* = 0.03). In 10–11 year-old pupils who received the ‘Nourish’ intervention compared with those that did not, their perceived cooking competence significantly increased (adjusted difference in mean = 3.21 95% CI 0.65,5.77; *P* = 0.02). No significant differences were observed for the ‘Engage’ intervention.

**Table 6:** Impact of the Nourish and Engage Interventions on Food Identification, Food Neophilia in both 6–7 and 10–11 year old age groups and Perceived Cooking Competence Scores in 10–11 year old age group

	6–7 year old pupils				10–11 year old pupils			
	Nourish	No Nourish	Engage	No Engage	Nourish	No Nourish	Engage	No Engage
**Food Identification Score**								
**N (baseline and follow-up responses)**	217	198	225	190	188	183	195	176
**Baseline Mean (SD)**	3.92 (1.31)	3.56 (1.66)	3.62 (1.60)	3.90 (1.36)	3.72 (0.92)	3.64 (0.97)	3.74 (1.77)	3.62 (1.03)
**Follow-up Mean (SD)**	4.43 (0.99)	4.33 (1.04)	4.37 (1.04)	4.41 (0.98)	3.82 (0.93)	3.87 (0.98)	3.78 (1.62)	3.91 (1.00)
**Adjusted diff. in mean 95% CI)**	0.00 (−0.19–0.20)	Reference	0.04 (−0.16–0.24)	Reference	−0.06 (− 0.36–0.24)	Reference	−0.17 (− 0.47–0.14)	Reference
***P*-value**	0.97		0.67		0.67		0.26	
**Food Neophilia Score**								
**N (baseline and follow-up responses)**	217	198	225	190	188	183	195	176
**Baseline Mean (SD)**	3.08 (1.37)	3.02 (1.49)	3.24 (1.43)	2.83 (1.40)	3.90 (1.78)	3.29 (1.82)	3.67 (1.77)	3.52 (1.88)
**Follow-up Mean (SD)**	3.42 (1.33)	3.10 (1.41)	3.41 (1.38)	3.08 (1.35)	4.02 (1.61)	3.58 (1.71)	3.83 (1.62)	3.77 (1.73)
**Adjusted diff. in mean 95% CI)**	0.27 (0.03–0.51)	Reference	0.07 (−0.16–0.30)	Reference	0.02 (−0.22–0.26)	Reference	−0.04 (− 0.26–0.18)	Reference
***P*-value**	0.03		0.51		0.89		0.70	
**Perceived Cooking Competence**								
**N (baseline and follow-up responses)**	–	–	–	–	188	183	195	176
**Baseline Mean (SD)**					25.29 (15.29)	25.78 (15.10)	25.89 (15.22)	25.14 (15.16)
**Follow-up Mean (SD)**					26.82 (15.21)	24.08 (15.60)	25.62 (14.97)	25.30 (15.99)
**Adjusted diff. in mean 95% CI)**					3.21 (0.65–5.77)	Reference	−0.69 (−3.34–1.95)	Reference
***P*-value**	–		–		0.02		0.58	

*P* value < 0.05 indicative of significance; N Number, *SD* Standard Deviation. In factorial analysis, the 2 main effects (Nourish compared with no nourish, and Engage compared with no engage) are investigated

## Discussion

This paper reports on Project Daire, a 4-arm multicomponent randomised controlled factorial design trial evaluating two food-based interventions in primary schools in Northern Ireland. Overall, results indicate that the ‘Nourish’ intervention, which aimed to alter the whole-school food environment and increase exposure to locally produced foods, produced more positive changes in emotional and behavioural wellbeing, food knowledge, cooking competence and dietary intake than the ‘Engage’ food-education intervention in primary school children in an economically disadvantaged area.

The average Total Difficulties Score at baseline in the current sample was higher than published normative average SDQ data on 10,438 UK children aged 5–15 years (6.7; SD 5.9) published by the Office for National Statistics [32]. This would suggest that children who participated in the current trial had higher levels of emotional and behavioural problems than other UK schoolchildren of a similar age and may be related to the fact that the trial recruited schools from one of the most economically deprived geographical areas within Northern Ireland [11]. The Strengths and Difficulties questionnaire has been previously used to identify ‘at risk’ children in order to target interventions [33] and the ‘Nourish’ intervention in the current study led to an improvement in emotional and behavioural wellbeing in these pupils post-intervention, compared with pupils who did not receive the ‘Nourish’ intervention. The improvement in Total Difficulties score observed resulted in better than average scores post-intervention, when compared with norm data (average score 6.44) [32]. In the UK Incredible Years Teacher Classroom Management mental health intervention, short-term improvements were also observed in Total Difficulties Score (5.5 (SD: 5.4)) in the intervention group compared with control group (6.2 (SD 6.2)) at 9 months, particularly in children who were already struggling with their mental health [34]. This may also help to explain the difference in level of improvement seen when results were analysed by gender in subgroup analyses. Males reported higher, on average, Total Difficulties than females in the current study, a trend also reported in the norm data [32], and may therefore have had more to gain from such an intervention [32, 34]. However, it should be noted that these subgroup analyses have reduced power. The magnitude of improvement in pupils with lower than average wellbeing at baseline in the Incredible Years Teacher Classroom Management intervention was similar to that seen in the current study [34].

The average health-related quality of life scores (KIDSCREEN-10) in the current sample, were below the average European KIDSCREEN-10 Norm data for the 8–11 year-old category (mean: 53.9) at both baseline and endpoint, indicating a poorer than average quality of life in this sample. In a publication which assessed childhood quality of life using the KIDSCREEN-10 across 15 European countries, children from poorer socio-economic backgrounds also reported poorer quality of life [35], which may reflect the lower socio-economic backgrounds of the pupils who participated in Project Daire. No significant differences were observed in health-related quality of life post interventions in main analyses of the current study although health-related quality of life increased in all groups. Within subgroup analyses, health-related quality of life in males randomised to Nourish significantly improved post intervention compared with those who did not receive Nourish. Health related quality of life scores were higher in males than in female pupils in the current study, which contrasts with higher scores reported in males in the data from 15 European countries [35]. Therefore, it is unclear why the Nourish intervention produced no significant changes in health-related quality of life overall, but may have been more beneficial to males than females, despite males reporting better quality of life overall. The SDQ measure was completed by teachers for each individual pupil, whereas pupils completed the KIDSCREEN-10 measure of health-related quality of life independently and therefore quality of completion may have varied. A process evaluation of these interventions may help elucidate some of these uncertainties.

A number of indications of changes in dietary behaviour were observed in children who received the ‘Nourish’ intervention and this was most apparent in the 10–11 year-old age group, compared with pupils who did not receive the intervention. The dietary changes were observed across multiple food groups and largely reflected the foods the pupils were exposed to during the intervention, although an increased likelihood of consuming fizzy drinks and chocolate at endpoint was also observed and these items were not provided as part of the ‘Nourish’ intervention. It is therefore not clear why consumption of these foods increased in schools randomised to ‘Nourish’ compared with those that were not. There were fewer indications of differences in dietary intake observed in pupils who received the ‘Engage’ intervention compared with those who did not in the 10–11 year old age group, although some indications of changes in dietary behaviour were observed. Systematic reviews on school food environment interventions which included ‘whole-school’ approaches such as competitive food/beverage standards, improved school meal standards and changes to food availability e.g. in vending machines or tuck shops, have also led to improvements in dietary intake [9, 36]. A 24-h dietary record was also collected in this study, but quality of data collected was poor and often incomplete and these data were not presented within this manuscript.

With regards to the educational outcomes, knowledge of vegetables in season and perceived cooking competence improved in 10–11 year-old children who had received the ‘Nourish’ school food environment intervention compared with those who did not, whereas the ‘Engage’ educational intervention was associated with an improved understanding of food labels in 10–11 year-old pupils. In a systematic review on ‘whole-school’ nutrition education interventions, improvements in food knowledge and willingness to try new foods was also observed, particularly in multicomponent interventions involving teachers and parents which were of adequate duration, age appropriate and incorporated environmental changes to impact knowledge [10]. These findings are therefore comparable to the current results which found that, overall, the whole-school food environment intervention was more effective than the educational intervention in achieving improvement in some of the dietary and knowledge outcomes. This is perhaps due to the impact of the school environmental changes on knowledge and awareness, which may not be as effective using nutrition education interventions alone [7].

Nutritional standards for school lunches have been in place in Northern Ireland since 2007 [37] and were extended to cover all foods served in schools in 2013 [38]. However, there are no formal systems in place to regularly monitor implementation of the standards [7]. As such, results from this trial suggest that a whole-school food environment intervention like ‘Nourish’ has the potential to support the effective implementation of school food standards via a ‘whole-school’ approach and may be an effective means of improving health and behaviour overall.

This trial has a number of strengths. Overall, the interventions were well received by all participating primary schools and no schools were lost to follow-up. To the best of our knowledge, this is the first time ‘whole-school’ educational and environmental interventions were developed for UK primary schools using a uniquely collaborative approach with schools and wider public and private stakeholders. The majority of previous work conducted has implemented either an environmental or educational approach rather than both [9, 10] and as most environmental interventions to date have focused on more limited food groups such as fruit and vegetable interventions, Project Daire incorporated a ‘whole-diet’ approach.

The study had a number of limitations. The interventions were planned to be 6-months in duration but, due to circumstances beyond the control of the research team, such as the requirement to fit flexibly within school timetables, intervention duration ranged from 2.5 months to 5 months. The entire process of data collection and intervention delivery lasted 6 months. It has been suggested that it can take a significant amount of time to achieve change in these types of interventions, and increased effectiveness has been reported in interventions lasting 6 months or longer in duration [10, 39, 40]. The study team worked with teachers to develop age-specific measures, where relevant, yet data collection in the younger age group in particular remained time-consuming. Upon reflection, the number of measures collected in future school-based trials could perhaps be reduced further to ensure data collection can be completed more efficiently within the context of a busy school day. Project Daire had a 2 × 2 factorial design and therefore was not powered to test for any interaction between interventions. The power calculation performed for the primary outcomes indicated that 1152 pupils were required to complete the study, but this incorporated a generous 20% drop-out rate. As no schools or pupils dropped-out of the study, only 960 pupils were therefore required to complete the study and in total, data were collected for 903 pupils. Although this should be considered a limitation, reassuringly, results from additional analyses which included the non-randomised Irish language schools supported the main results with regards to the primary outcomes.

Due to the nature of the school-based interventions, which were largely delivered by the schools themselves, some variation in selection and implementation of the various intervention elements across schools and teachers was inevitable. Process evaluation data were collected according to MRC framework for process evaluation of complex interventions [41] and these aspects will be thoroughly explored and published separately. It will be of particular interest to examine the extent of the implementation of delivery of the ‘Engage’ intervention, which appeared to be less effective than the ‘Nourish’ intervention in the present study, to further elucidate potential reasons for this. Another consideration is the sustainability of a school-based interventions such as those implemented in the current study. Project Daire has demonstrated the feasibility of such school-based interventions in the short term, but it would be of interest to explore the feasibility of long-term implementation and the potential model for sustainable delivery, for example, through a public-private partnership.

In conclusion, our results suggest that modifying the whole-school food environment in UK primary schools has a positive effect on children’s wellbeing, knowledge about food and dietary intake in those most at need within economically deprived regions. These results are especially important in light of the pandemic-related school closures worldwide and the impact of COVID-19 on levels of food insecurity. These data reflect the increasing recognition that interventions should focus on the wider school food environment in addition to educational aspects, to ensure a ‘whole-school’ approach. In addition to the evaluation of effectiveness of such interventions in different regions, future work should explore cost-effectiveness and sustainability issues.

## Supplementary Information

**Additional file 1. **CONSORT Checklist for Cluster Randomised Controlled Trial.

**Additional file 2. **TIDieR Checklist .

**Additional file 3. **Intervention Components.

**Additional file 4. **Additional School Analysis.

**Additional file 5.** Additional Outcomes.

**Additional file 6.** Gender Analyses.

**Additional file 7.** Logic Model.

## Data Availability

The datasets used and/or analysed during the current study are available from the corresponding author on reasonable request.
