# Supplementary material for: Food environment intervention improves food knowledge, wellbeing and dietary habits in primary school children: Project Daire, a randomised-controlled, factorial design cluster trial
Source: Int J Behav Nutr Phys Act. 2021 Feb 4;18:23. doi: 10.1186/s12966-021-01086-y (PMC7859905; doi:10.1186/s12966-021-01086-y)
Supplement: Supplementary file 3 — Additional file 3. Intervention Components. [file 12966_2021_1086_MOESM3_ESM.docx]

**Additional File 3: Listing of all available ‘Nourish’ and ‘Engage’ intervention components.**

**Table 1. ‘Engage’ Intervention Lesson Plans by Topic**

|  | **Topics** | | |
| --- | --- | --- | --- |
|  | **Farm to Fork** | **Pleasure on a Plate** | **Food Futures** |
| **Lesson Plans** | Food Chain | Identification of a range of foods | Making the same food different |
|  | Food Source to Product | How do we Taste? | New Product Development |
|  | High Tech Agriculture/Food Production | Healthy Choices/Food Swaps | Marketing |
|  | Local versus Imported | Buying/Cost | Advertising |
|  | Growing Food | Preparing | Businesses need people with a variety of skills |
|  | Welfare | Food Labels | Showcasing ideas in schools |
|  | Animal Feeding | Portion Size |  |
|  | Sustainability | Diet and Health |  |
|  | Climate Change | Physical Activity/Broader Lifestyle |  |

**Table 2. ‘Engage’ Intervention Topics and Associated Learning Intentions**

|  | **Food Futures**  **(each lesson 30 minutes, can be combined as 1-hour lesson)** | **Farm to Fork**  **(1-hour lessons)** | **Pleasure on a Plate**  **(1-hour lessons)** |
| --- | --- | --- | --- |
| **Topic Overview** | Lesson 1: Favourite Food (making the same food different, new product development) | Lesson 1: Animal Welfare (welfare, climate change, sustainability, local food production and culture) | Lesson 1: Sensory Scientist (how do we taste) |
|  | Lesson 2: Food Ideas (making the same food different, new product development) | Lesson 2: Johnny Loves Milk (food source to product, food chain, high tech agriculture, animal feeding) | Lesson 2: Portion Size (portion size, lifestyle) |
|  | Lesson 3: In the Restaurant (making the same food different, new product development, showcasing ideas in school) | Lesson 3: Food Scribblers (food source to product, food chain) | Lesson 3: Seasonality (local versus imported food, seasonality, careers, buying/cost) |
|  | Lesson 4: Building Ideas (making the same food different, new product development) | Lesson 4: Food Stories (local food production and culture, careers) | Lesson 4: Growing (identification of a range of foods, growing food) |
|  | Lesson 5: Marketing (marketing, advertising, businesses need people with a variety of skills and jobs) | Lesson with classroom visitor (high tech agriculture, food source to product) | Lesson with classroom visitor (food labels, portion size, healthy choices) |
|  | Lesson 6: Advertising (marketing, advertising, businesses need people with a variety of skills and jobs) | Lesson with classroom visitor (food source to product, food chain) | Lesson with classroom visitor (physical activity) |
|  | Lesson 7: Great Teams (marketing, advertising, businesses need people with a variety of skills and jobs) |  |  |
|  | Lesson 8: Business Planning (marketing, advertising, businesses need people with a variety of skills and jobs) |  |  |
|  | School Showcase |  |  |
| **Visit Options** | Mobile farm to school, visit to a range of local food industry partners premises/factories. | | |

**Table 3. ‘Nourish’ Intervention Components**

| **Nourish Intervention Component** | **Description** |
| --- | --- |
| **Healthy Snack Delivery** | Several of the participating food industry partners provided milk, fruit snack packs and a variety of breads to pupils participating in the Nourish intervention twice per week during the school day. Selected snacks adhered to the Northern Ireland Food in Schools Policy (2013) (1). |
| **Enhancement of school canteen/dining area** | Provision of tablecloths, centre pieces, bunting, posters on where food comes from and healthy eating and menu boards for school canteen/dining area with the aim of enhancing the school dining experience and creating a café-style atmosphere |
| **Enhanced presentation of healthy foods in school canteen/dining area** | Provision of a pack containing serve ware such as serving platters, tiered stands and individual re-usable serving cups and paper straws, salad trolley and equipment for cutting foods so that fruit, vegetables and healthy snacks could be presented in creative ways in the school environment to encourage consumption. |
| **Tasting Day** | Pupils had the opportunity to attend an interactive ‘Tasting Day’ event held at a Higher Education facility. Participating food industry partners showcased their produce and encouraged pupils to taste new foods and participate in hands on food preparation activities. Pupils received a ‘food passport’ to record the new foods they had tasted and were provided with a goody bag and local food products to take home |
| **Cookery equipment and recipes** | Recipe book developed by the research team to encourage balanced diet and interest in preparation of food. Recipe book was developed for use in the school setting e.g. school cookery activities and pupils were given a printed copy to take home to encourage home cookery activities with parents/ guardians The recipes utilised local foods and included recipes for children, taken from the Council for the Curriculum, Examinations and Assessment (CCEA) ‘Growing for the Future’ resource (<http://ccea.org.uk/growing>) as well as recipes for adults. Schools were provided with a range of cookery equipment e.g. mixing bowls, scales, graters, wooden spoons, rolling pins, to support the cookery activities. |
| **Sensory Education** | Schools were provided with a sensory education resource adapted for the ‘Nourish’ intervention from the Flavour School programme produced by Flavour School UK ([www.flavourschool.org.uk](http://www.flavourschool.org.uk)). The resource outlined 5 fun and simple activities to help children learn about the senses, taste and flavour, and to encourage them to try new foods. |
| **School Food Event** | Food industry partners provision of food at food themed event |
| **Working with staff to ensure optimal school food policies** | School senior management were sent a document containing recommendations for changes that could be made to school food policies |

References:

1. Department of Education and Department of Health, Social Services and Public Safety. Food in Schools Policy 2013 [Available from: https:www.education-ni.gov.uk/sites/default/files/publications/de/healthy-%20food-for-healthy-outcomes---food-in-schools-policy---english-version.pdf. Accessed: May 3^rd^ 2020.
