# Supplementary material for: Food environment intervention improves food knowledge, wellbeing and dietary habits in primary school children: Project Daire, a randomised-controlled, factorial design cluster trial
Source: Int J Behav Nutr Phys Act. 2021 Feb 4;18:23. doi: 10.1186/s12966-021-01086-y (PMC7859905; doi:10.1186/s12966-021-01086-y)
Supplement: Supplementary file 4 — Additional file 4. Additional School Analysis. [file 12966_2021_1086_MOESM4_ESM.docx]

**Additional File 4: Results for the Primary Outcomes for the Nourish Intervention with All Schools Included (Including Irish Language Schools)**

**Table 1. Impact of the Nourish Intervention on Strengths and Difficulties Scales and KIDSCREEN-10 Rasch Parameter Estimates and International T-Values (All schools including Irish Language Schools and all age groups).**

|  | **Nourish** | **No Nourish** |
| --- | --- | --- |
| **Emotional Problems (SDQ)**  **N (baseline and follow-up responses)**  **Baseline Mean (SD)**  **Follow-up Mean (SD)**  **Adjusted diff. in mean (95% CI)** | 344  1.99 (2.42)  1.56 (2.20)  -0.34 (-0.75) | 254  2.08 (2.57)  1.97 (2.49)  Reference |
| **P-value** | 0.08 | |
| **Conduct Problems Scale (SDQ)**  **N (baseline and follow-up responses)**  **Baseline Mean (SD)**  **Follow-up Mean (SD)**  **Adjusted diff. in mean (95% CI)** | 340  1.15 (1.97)  0.95 (1.66)  -0.27 (-0.54- -0.00) | 255  1.18 (1.99)  1.24 (1.94)  Reference |
| **P-value** | 0.04 | |
| **Hyperactivity Scale (SDQ)**  **N (baseline and follow-up responses)**  **Baseline Mean (SD)**  **Follow-up Mean (SD)**  **Adjusted diff. in mean (95% CI)** | 347  3.43 (1.97)  3.02 (2.99)  -0.25 (-0.73- 0.23) | 260  3.09 (1.98)  3.02 (3.11)  Reference |
| **P-value** | 0.28 | |
| **Peer Problems Scale (SDQ)**  **N (baseline and follow-up responses)**  **Baseline Mean (SD)**  **Follow-up Mean (SD)**  **Adjusted diff. in mean (95% CI)** | 345  1.24 (1.77)  0.95 (1.51)  -0.26 (-0.54- 0.01) | 256  1.28 (1.63)  1.24 (1.77)  Reference |
| **P-value** | 0.06 | |
| **ProSocial Scale (SDQ)**  **N (baseline and follow-up responses)**  **Baseline Mean (SD)**  **Follow-up Mean (SD)**  **Adjusted diff. in mean 95% CI)** | 344  7.51 (2.63)  7.70 (2.59)  -0.02 (-0.71- 0.67) | 255  7.73 (2.49)  7.88 (2.41)  Reference |
| **P-value** | 0.94 | |
| **Total Difficulties Score (SDQ)**  **N (baseline and follow-up responses)**  **Baseline Mean (SD)**  **Follow-up Mean (SD)**  **Adjusted diff. in mean 95% CI)** | 246  7.39 (6.88)  6.94 (6.66)  -1.16 (-2.32- 0.00) | 336  7.96 (7.18)  7.32 (6.89)  Reference |
| **P-value** | 0.05 | |
| **General Health Related Quality of Life Index Rasch Parameter Estimates (KIDSCREEN-10)**  **N (baseline and follow-up responses)**  **Baseline Mean (SD)**  **Follow-up Mean (SD)**  **Adjusted diff. in mean 95% CI)** | 496  1.05 (1.09)  1.25 (1.16)  0.01 (-0.20-0.23) | 381  1.07 (1.08)  1.25 (1.16)  Reference |
| **P-value** | 0.90 | |
| **General Health Related Quality of Life Index International T Values (KIDSCREEN-10)**  **N (baseline and follow-up responses)**  **Baseline Mean (SD)**  **Follow-up Mean (SD)**  **Adjusted diff. in mean 95% CI)** | 496  50.46 (11.24)  48.51 (10.55)  0.12 (-1.99- 2.23) | 381  50.40 (11.26)  48.60 (10.51)  Reference |
| **P-value** | 0.90 | |

*P value <0.05 indicative of significance; N: Number; SD: Standard Deviation.*
