# Supplementary material for: Food environment intervention improves food knowledge, wellbeing and dietary habits in primary school children: Project Daire, a randomised-controlled, factorial design cluster trial
Source: Int J Behav Nutr Phys Act. 2021 Feb 4;18:23. doi: 10.1186/s12966-021-01086-y (PMC7859905; doi:10.1186/s12966-021-01086-y)
Supplement: Supplementary file 5 — Additional file 5. Additional Outcomes. [file 12966_2021_1086_MOESM5_ESM.docx]

| **Study Outcomes** | **Information and Development** | **Administration** | **Scoring** |
| --- | --- | --- | --- |
| 24-hour Prospective Dietary Record | This measure was based on a dietary measure for children that had been previously developed (1). The original measure allows children to both draw and write down their dietary intake over the previous 24-hour period. After consultation with primary school teachers and after consideration of the difficulties young children may experience when recalling their dietary intake over the previous 24-hour period, the reference period was amended to make it prospective rather than retrospective to allow researchers to assist children with recording what they consumed over the school day. | Administered to all pupils. The questionnaire was split into two sections: ‘school’ and ‘home’ over the 24-hour period. Researchers assisted children with completion of the school section whilst they were present during data collection. Children were asked to take the ‘home’ section of the questionnaire home and parents and teachers assisted with completion of the remainder of the 24-hour period. | Quality of the dietary intake reporting will be recorded and assessed e.g. completion rates of both ‘school’ and ‘home’ dietary sections. Dietary intake over the 24-hour period will then be entered directly into dietary analyses software and average daily intakes of a range of food groups, macronutrients, micronutrients, vitamins and minerals will be assessed. |
| Attitudes | A Health Attitudes and Behaviour measure which was previously used in a Northern Ireland schoolchildren population as part of the Young Hearts Project (2) | Administered to 10-11 year old pupils only | An 11-item instrument assessing attitudes towards importance of various health behaviours 'very important', 'quite important', 'not important'. |
| Food Jobs/Career Aspirations | Assessed career aspirations and knowledge of food-related jobs. | Administered to all pupils at baseline and endpoint | Open-ended questions asking about career aspirations and assessing knowledge of careers related to food. |
| Trust | Questionnaire to assess level of trust in local food products. Developed by research team. | Administered to 10-11 year old pupils only. | This is a 10-item instrument answered on a 5-point scale (definitely disagree, disagree, not sure, agree, definitely agree). |
| Exposure to/Experience with Agri-Food | Measure designed to characterise pupils in terms of previous experience of agri-food | Administered to all pupils at baseline only | Categorical responses. |
| School Food Environment Observation | Observation proforma was based on the published Health Promotion Agency document ‘Improving the dining experience in schools’ (3) | Completed for all schools by study researcher. | Researcher observations on school food environment including school food policies, school canteen environment etc. |
| School Food Environment Questionnaire | Measure was adapted from a school environment survey developed for the Health Behaviour in School-Aged Children Survey (HBSC) conducted in Wales (4, 5, 6). | Administered to school principals or other relevant contacts e.g. Vice Principal or nominated teacher. | Categorical and open-ended responses |
| Process Evaluation | A process evaluation protocol was developed based on the Medical Research Council (MRC) published guidance for process evaluation of complex interventions (7). | Combination of research observation of various elements of the interventions, record keeping of intervention component selection and questionnaires designed for teachers of the 6-7 and 10-11 year old classes. | As per Medical Research Council guidance for process evaluations of complex interventions (7). |

**Additional File 5: Study outcomes not presented in the current paper.**

Please note educational attainment data for the pupils was obtained from a small number of the schools to test whether it was feasible to collect these data.

Please note: analyses of these secondary outcome measures will be published separately.

**References**

1. Moore GF, Tapper K, Murphy S, Clark R, Lynch R, Moore L. Validation  of a self-completion  measure of breakfast foods, snacks and fruits and vegetables consumed by 9- to 11- year old schoolchildren. EJCN. 2007, 61:420-430.
2. Gallagher AM, Savage JM, Murray LJ, Davey Smith G, Young IS, Robson PJ, Neville CE, Cran G, Strain JJ, Boreham CA. A longitudinal study through adolescence to adulthood: the Young Hearts Project, Northern Ireland. Public Health. 2002, 116: 332-340.
3. Health Promotion Agency. Improving the dining experience in schools 2009. [Available from: <https://www.publichealth.hscni.net/sites/default/files/Dining%20Experience%2009_10.pdf>] Accessed: 28.01.20.
4. Moore GF, Audrey S., Barker M, Bond L, Bonell C, Hardeman W. et al. Process evaluation of complex intervention: medical research council guidance. BMJ. 2015, 350: h1258.
5. Davison J. Wellbeing in schools (WiSe) Survey 2018. School Climate Questionnaire.
6. Northern Ireland Statistics and Research Agency. Young Persons Behaviour and Attitudes Survey. Technical Report 2016. [Available from: <https://www.health-ni.gov.uk/publications/young-persons-behaviour-and-attitudes-survey-2016>] Accessed: May 12^th^ 2020.
7. Moore GF, Littlecott HJ, Fletcher A. et al. (2015). Variations in schools’ commitment to health and implementation of health improvement activities: a cross-sectional study of secondary schools in Wales. BMC Public Health. 2015, 16, 138.
