# Supplementary material for: Food environment intervention improves food knowledge, wellbeing and dietary habits in primary school children: Project Daire, a randomised-controlled, factorial design cluster trial
Source: Int J Behav Nutr Phys Act. 2021 Feb 4;18:23. doi: 10.1186/s12966-021-01086-y (PMC7859905; doi:10.1186/s12966-021-01086-y)
Supplement: Supplementary file 6 — Additional file 6. Gender Analyses. [file 12966_2021_1086_MOESM6_ESM.docx]

**Additional File 6: Gender Analyses**

**Impact of the Nourish and Engage Interventions on Strengths and Difficulties (SDQ) and KIDSCREEN-10 Rasch Parameter Estimates and International T-Values in Females**

|  | **Nourish** | **No Nourish** | **Engage** | **No Engage** |
| --- | --- | --- | --- | --- |
| **Emotional Problems (SDQ)**  **N (baseline and follow-up responses)**  **Baseline Mean (SD)**  **Follow-up Mean (SD)**  **Adjusted diff. in mean (95% CI)** | 131  1.93 (2.48)  1.39 (1.97)  -0.35 (-0.84- 0.12) | 128  2.17 (2.62)  1.89 (2.51)  Reference | 165  2.04 (2.59)  1.80 (2.41)  0.44 (-0.00-0.90) | 94  2.07 (2.51)  1.37 (1.96)  Reference |
| **P-value** | 0.13 | | 0.05 | |
| **Conduct Problems Scale (SDQ)**  **N (baseline and follow-up responses)**  **Baseline Mean (SD)**  **Follow-up Mean (SD)**  **Adjusted diff. in mean (95% CI)** | 129  0.78 (1.87)  0.62 (1.51)  -0.11 (-0.36-0.12) | 128  0.60 (1.26)  0.63 (1.33)  Reference | 162  0.73 (1.78)  0.58 (1.42)  -0.19 (-0.44-0.06) | 95  0.63 (1.24)  0.70 (1.41)  Reference |
| **P-value** | 0.34 | | 0.15 | |
| **Hyperactivity Scale (SDQ)**  **N (baseline and follow-up responses)**  **Baseline Mean (SD)**  **Follow-up Mean (SD)**  **Adjusted diff. in mean (95% CI)** | 131  2.13 (1.87)  1.86 (2.56)  -0.08 (-0.47-0.27) | 132  1.98 (1.25)  1.84 (2.43)  Reference | 167  1.97 (1.76)  1.74 (2.41)  -0.10 (-0.52-0.31) | 96  2.21 (1.24)  2.03 (2.62)  Reference |
| **P-value** | 0.62 | | 0.59 | |
| **Peer Problems Scale (SDQ)**  **N (baseline and follow-up responses)**  **Baseline Mean (SD)**  **Follow-up Mean (SD)**  **Adjusted diff. in mean (95% CI)** | 130  1.03 (1.52)  0.74 (1.27)  -0.26 (-0.56-0.04) | 130  0.95 (1.39)  0.94 (1.62)  Reference | 167  1.11 (1.53)  0.88 (1.57)  -0.10 (-0.33-0.12) | 93  0.78 (1.29)  0.76 (1.23)  Reference |
| **P-value** | 0.08 | | 0.33 | |
| **ProSocial Scale (SDQ)**  **N (baseline and follow-up responses)**  **Baseline Mean (SD)**  **Follow-up Mean (SD)**  **Adjusted diff. in mean 95% CI)** | 129  8.28 (2.31)  8.49 (2.02)  -0.01 (-0.97-0.95) | 129  8.44 (2.17)  8.59 (2.05)  Reference | 163  8.57 (3.21)  8.58 (2.09)  -0.23 (-1.10-0.63) | 95  8.00 (2.08)  8.48 (1.94)  Reference |
| **P-value** | 0.98 | | 0.56 | |
| **Total Difficulties Score (SDQ)**  **N (baseline and follow-up responses)**  **Baseline Mean (SD)**  **Follow-up Mean (SD)**  **Adjusted diff. in mean 95% CI)** | 128  5.91 (6.56)  4.57 (5.37)  -0.95 (-2.04- 0.14) | 123  5.61 (5.85)  5.31 (5.79)  Reference | 160  5.76 (6.42)  4.97 (5.78)  0.13 (-0.01-1.17) | 91  5.78 (5.86)  4.87 (5.22)  Reference |
| **P-value** | 0.08 | | 0.79 | |
| **General Health Related Quality of Life Index Rasch Parameter Estimates (KIDSCREEN-10)**  **N (baseline and follow-up responses)**  **Baseline Mean (SD)**  **Follow-up Mean (SD)**  **Adjusted diff. in mean 95% CI)** | 205  1.06 (1.05)  1.37 (1.14)  -0.01 (-0.28-0.26) | 189  1.14 (1.13)  1.42 (1.15)  Reference | 222  1.11 (1.11)  1.37 (1.18)  -0.05 (-0.33-0.22) | 172  1.09 (1.07)  1.42 (1.10)  Reference |
| **P-value** | 0.90 | | 0.66 | |
| **General Health Related Quality of Life Index International T Values (KIDSCREEN-10)**  **N (baseline and follow-up responses)**  **Baseline Mean (SD)**  **Follow-up Mean (SD)**  **Adjusted diff. in mean 95% CI)** | 205  48.6 (10.2)  51.6 (11.0)  -0.06 (-2.74-2.60) | 189  49.4 (10.9)  52.0 (11.2)  Reference | 222  49.01 (10.76)  51.61 (11.48)  -0.55 (-3.25-2.15) | 172  48.87 (10.37)  52.09 (10.69)  Reference |
| **P-value** | 0.95 | | 0.66 | |

*P value <0.05 indicative of significance; N: Number; SD: Standard Deviation.*  *In factorial analysis, the 2 main effects (Nourish compared with no nourish, and Engage compared with no engage) are investigated*

**Impact of the Nourish and Engage Interventions on Strengths and Difficulties (SDQ) and KIDSCREEN-10 Rasch Parameter Estimates and International T-Values in Males**

|  | **Nourish** | **No Nourish** | **Engage** | **No Engage** |
| --- | --- | --- | --- | --- |
| **Emotional Problems (SDQ)**  **N (baseline and follow-up responses)**  **Baseline Mean (SD)**  **Follow-up Mean (SD)**  **Adjusted diff. in mean (95% CI)** | 148  2.16 (2.60)  1.91 (2.48)  -0.25 (-0.65- 0.13) | 126  1.99 (2.52)  2.05 (2.48)  Reference | 157  2.33 (2.76)  2.24 (2.61)  0.24 (-0.17-0.66) | 117  1.75 (2.23)  1.61 (2.24)  Reference |
| **P-value** | 0.17 | | 0.23 | |
| **Conduct Problems Scale (SDQ)**  **N (baseline and follow-up responses)**  **Baseline Mean (SD)**  **Follow-up Mean (SD)**  **Adjusted diff. in mean (95% CI)** | 149  1.46 (1.98)  1.36 (1.90)  -0.27 (-0.56-0.02) | 127  1.77 (2.39)  1.86 (2.25)  Reference | 157  1.62 (2.09)  1.57 (2.03)  -0.07 (-0.36-0.28) | 119  1.58 (2.30)  1.63 (2.14)  Reference |
| **P-value** | 0.07 | | 0.63 | |
| **Hyperactivity Scale (SDQ)**  **N (baseline and follow-up responses)**  **Baseline Mean (SD)**  **Follow-up Mean (SD)**  **Adjusted diff. in mean (95% CI)** | 150  4.36 (1.98)  4.21 (3.16)  -0.11 (-0.58-0.33) | 128  4.23 (2.39)  4.25 (3.27)  Reference | 158  4.39 (2.09)  4.17 (3.25)  -0.27 (-0.72-0.19) | 120  4.19 (2.29)  4.30 (3.17)  Reference |
| **P-value** | 0.60 | | 0.25 | |
| **Peer Problems Scale (SDQ)**  **N (baseline and follow-up responses)**  **Baseline Mean (SD)**  **Follow-up Mean (SD)**  **Adjusted diff. in mean (95% CI)** | 149  1.51 (1.91)  1.30 (1.79)  -0.17 (-0.48- 0.15) | 126  1.62 (1.79)  1.55 (1.86)  Reference | 158  1.55 (1.71)  1.36 (1.83)  -0.08 (-0.43-0.24) | 117  1.58 (2.04)  1.48 (1.81)  Reference |
| **P-value** | 0.27 | | 0.59 | |
| **ProSocial Scale (SDQ)**  **N (baseline and follow-up responses)**  **Baseline Mean (SD)**  **Follow-up Mean (SD)**  **Adjusted diff. in mean 95% CI)** | 149  6.74 (2.72)  6.81 (2.86)  -0.15 (-0.82-0.50) | 126  7.01 (2.60)  7.15 (2.53)  Reference | 157  6.81 (2.74)  7.03 (2.80)  0.25 (-0.37-0.90) | 118  6.94 (2.55)  6.87 (2.61)  Reference |
| **P-value** | 0.61 | | 0.38 | |
| **Total Difficulties Score (SDQ)**  **N (baseline and follow-up responses)**  **Baseline Mean (SD)**  **Follow-up Mean (SD)**  **Adjusted diff. in mean 95% CI)** | 146  9.50 (7.67)  8.86 (7.04)  -0.75 (-1.36- -0.12) | 123  9.68 (7.49)  9.76 (7.29)  Reference | 156  9.91 (7.69)  9.41 (7.35)  -0.26 (-0.92-0.36) | 113  9.13 (7.42)  9.08 (6.91)  Reference |
| **P-value** | 0.02 | | 0.38 | |
| **General Health Related Quality of Life Index Rasch Parameter Estimates (KIDSCREEN-10)**  **N (baseline and follow-up responses)**  **Baseline Mean (SD)**  **Follow-up Mean (SD)**  **Adjusted diff. in mean 95% CI)** | 197  1.09 (1.08)  1.35 (1.18)  0.23 (0.13-0.46) | 192  0.99 (1.04)  1.08 (1.14)  Reference | 198  1.03 (1.01)  1.21 (1.11)  -0.37 (-0.25-0.17) | 191  1.06 (1.10)  1.22 (1.16)  Reference |
| **P-value** | 0.03 | | 0.70 | |
| **General Health Related Quality of Life Index International T Values (KIDSCREEN-10)**  **N (baseline and follow-up responses)**  **Baseline Mean (SD)**  **Follow-up Mean (SD)**  **Adjusted diff. in mean 95% CI)** | 197  48.93 (10.53)  51.40 (11.46)  2.31 (0.13-4.47) | 192  47.93 (10.06)  48.77 (11.10)  Reference | 198  48.28 (9.95)  50.04 (11.41)  -0.37 (-2.48-1.73 ) | 191  48.61 (10.66)  50.17 (11.30)  Reference |
| **P-value** | 0.03 | | 0.70 | |

*P value <0.05 indicative of significance; N: Number; SD: Standard Deviation.*  *In factorial analysis, the 2 main effects (Nourish compared with no nourish, and Engage compared with no engage) are investigated*
