# Supplementary figures and images for: Food environment intervention improves food knowledge, wellbeing and dietary habits in primary school children: Project Daire, a randomised-controlled, factorial design cluster trial
Source: Int J Behav Nutr Phys Act. 2021 Feb 4;18:23. doi: 10.1186/s12966-021-01086-y (PMC7859905; doi:10.1186/s12966-021-01086-y)

**Additional File 7: Logic Model**


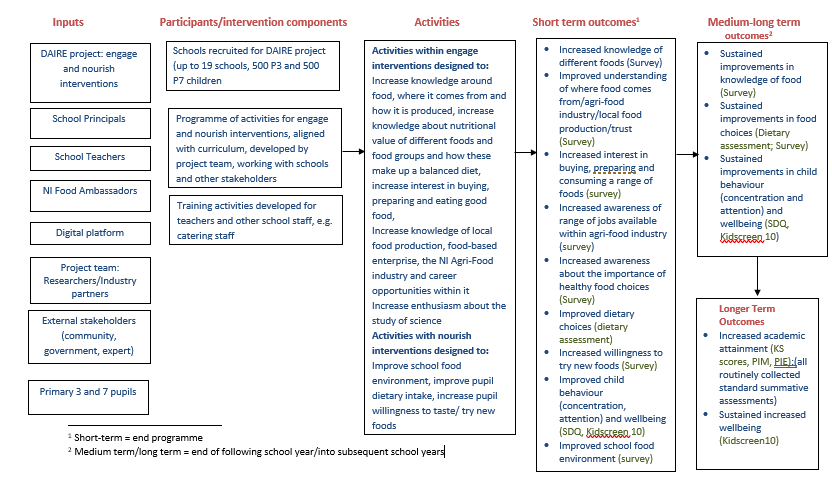

Supplement: Supplementary file 7 — Additional file 7. Logic Model. [file 12966_2021_1086_MOESM7_ESM.docx]
